# Do We Need Distinct Pediatric Classification Criteria for Rheumatic Diseases That Affect Both Children and Adults?

**DOI:** 10.1002/art.43058

**Published:** 2025-01-05

**Authors:** Coziana Ciurtin, Marija Jelusic, Seza Ozen

**Affiliations:** ^1^ Centre for Adolescent Rheumatology University College London London United Kingdom; ^2^ University Hospital Center Zagreb, University of Zagreb School of Medicine Zagreb Croatia; ^3^ Hacettepe University Faculty of Medicine Ankara Türkiye

## Introduction

Rheumatic diseases (RMDs) represent a diverse group of conditions that can affect individuals of any age. Although pediatric and adult rheumatologists develop specialized expertise in the recognition, diagnosis, and management of these diseases, individuals diagnosed with RMDs at distinct stages in their life face age and developmentally distinct challenges, which necessitate a comprehensive approach to health care. This approach must encompass coordinated and integrated care, including effective transition protocols for adolescents, as well as access to high‐quality research at every stage in life. Developmental factors play a key role in shaping the immune system, resulting in age‐related differences in disease risk, outcomes, and treatment responses, emphasizing the necessity for age‐tailored approaches in both research and clinical care. This is particularly crucial because genetic predispositions, environmental influences, and socioeconomic conditions can heavily impact RMD presentation and outcomes.

The need for improved evidence‐based health care in rheumatology requires high‐quality, multicenter studies with suitable geographic and ethnic representation across the lifespan. Accurate classification criteria help identify homogeneous disease populations, which is vital for advancing the understanding of the disease pathogenesis and for the development and testing of tailored therapeutic strategies. Moreover, adequate classification criteria can enhance the identification of disease subtypes with different outcomes and treatment responses, paving the way for more individualized management approaches.

Although there are similarities in the presentation and management of RMDs between distinct age groups such as children and adults or younger and older adults, significant differences in pathogenesis, clinical phenotype, disease progression, and outcomes often necessitate age‐specific classification criteria. However, this distinction has only been made for children rather than adults with certain RMDs, using an arbitrary age cut‐off of 16 or 18 years. Two main strategies have been employed in the classification of pediatric RMDs: one involves adapting adult classification criteria for use in pediatric populations with similar disease phenotypes, whereas the other focuses on developing and validating criteria specifically tailored to children.

A harmonized classification across the lifespan has practical implications. Clinicians recognize that epidemiologic differences between RMDs affecting children and adults should not significantly impact the performance of shared classification criteria across the lifespan. However, epidemiologic differences could be underpinned by unique genetic traits and immune characteristics of children compared to adults, reflected in distinct disease susceptibility as well as more severe trajectories for RMDs that emerge in childhood, aspects that may not be optimally addressed by a harmonized classification and research approach across the lifespan.

Conversely, there are phenotypical similarities between children and adults that span arbitrary age boundaries, which should support the use of similar classification criteria, as well as differences in clinical phenotype and pathogenesis between younger and older individuals affected by the same disease, which may not be reflected in the existent adult classification criteria. These age‐related variations can be attributed to factors beyond genetics or immune system maturation and aging, including alterations in the microbiome and environmental exposures that contribute to age‐dependent RMD patterns, all relevant for the selection of homogeneous disease populations for research purposes.

Because various approaches to classify RMDs in pediatric and adult rheumatology exist, we aim to evaluate their relevance and limitations and propose, wherever possible, to promote classification criteria that reflect shared clinical presentation and pathogenic mechanisms across age‐corresponding RMD phenotypes while also accounting for developmental and immunologic factors that may influence disease trajectories, when relevant.

## Classification of chronic inflammatory arthritis across the lifespan

Using the same classification criteria for children and adults with RMDs could harmonize research across age groups and facilitate the inclusion of both pediatric and adult populations in clinical trials. This approach could potentially expedite the approval and availability of new therapeutic options. However, standardizing the classification of RMDs is highly dependent on the particularities of the diagnostic label used in children compared with adults, which may reflect more or less heterogeneous disease phenotypes. For example, rheumatoid arthritis (RA) reflects a relatively homogeneous inflammatory arthritis phenotype in adults, whereas juvenile idiopathic arthritis (JIA) is an umbrella diagnosis in children, encompassing several subtypes of childhood arthritis characterized by heterogeneous pathogenesis and clinical presentation.

The International League of Associations for Rheumatology (ILAR) criteria,^1^ commonly used for classifying JIA, have several limitations. These include arbitrary age and joint count cut‐offs as well as mutually exclusive features that can complicate the classification for individuals who exhibit overlapping signs and symptoms. Moreover, the ILAR classification criteria do not address the genetic or molecular similarities among different JIA subtypes,^2^ which can impact the adequate treatment selection and accurate risk‐stratification of individuals with JIA. The ILAR subgroup of nonsystemic JIA that shares clear genetic and pathogenic mechanisms with their adult corresponding phenotype is the seropositive poly‐JIA group, which often affects adolescent girls and resembles seropositive RA. The pathogenesis of early onset forms of chronic arthritis observed in young patients is less well‐defined.

The Paediatric Rheumatology International Trials Organisation (PRINTO) proposed a novel classification system for JIA, categorizing the condition into four distinct subtypes.^3^ This classification introduces the age cut‐off of 18 years, in alignment with the World Health Organization definition of childhood age‐span, as well as an additional category for early‐onset JIA with positive antinuclear antibodies, which the ILAR criteria^1^ do not cover. The PRINTO criteria^3^ also suggest renaming enthesitis‐related arthritis as enthesitis‐ or spondylitis‐related JIA, recognizing its pathogenic similarities with ankylosing spondylitis. However, the exclusion of juvenile psoriatic arthritis (JPsA) from the PRINTO criteria poses challenges, especially because there are therapies specifically licensed for JPsA.^4^

A study exploring the overlap between the ILAR and PRINTO criteria in a large UK cohort found that nearly 70% of young people with JIA could not be classified into any of the named PRINTO categories.^5^ This significant discrepancy highlights the limitations of current JIA classification systems and underscores the need for further refinement and validation across diverse populations. Although anti‐citrullinated peptide antibodies are important for diagnosing and predicting outcomes in RA, and despite its genetic and phenotypic similarities to seropositive polyarticular JIA, these antibodies are not included in either the ILAR or PRINTO JIA classification criteria.

The recent decision by the Paediatric Rheumatology European Society (PReS) and the EULAR to recognize systemic JIA and adult‐onset Still disease as manifestations of the same condition, now termed Still disease, is a noteworthy development.^6^ This consensus is based on shared pathogenic mechanisms, clinical presentations, and treatment strategies, underscoring the importance of a unified approach to disease classification.

## Classification of systemic connective tissue diseases, vasculitides, and autoinflammatory conditions across the lifespan

These RMDs share the same pathogenesis and have rather consistent features throughout the lifespan. Systemic lupus erythematosus (SLE), with the exception of monogenic lupus, is characterized by the same type of organ and systems involvement in children and adults, with recognized differences in the prevalence of certain manifestations: a higher proportion of individuals with childhood‐onset SLE (cSLE) experience systemic manifestations, lupus nephritis, and central nervous system involvement compared to adults with SLE according to various cohort studies. These differences may reflect the increased genetic burden in children as well as challenges in accessing adult SLE treatment worldwide and poorer compliance to medication in adolescence as potential factors.

Distinct classification criteria for cSLE have not been deemed necessary. Differences in presentation and severity of cSLE were adequately captured at the disease onset by the adult criteria because they are not underpinned by differences in pathogenesis between cSLE and adult‐onset SLE. The American College of Rheumatology (ACR)^7^ and the Systemic Lupus International Collaborating Clinics criteria,^8^ commonly used to support SLE diagnosis, have been validated in both adult and pediatric populations, with recent updates improving their applicability across age groups.^9^ The ACR and EULAR proposed novel classification criteria in 2019, subsequently validated in adult SLE populations, were found to have satisfactory performance in cSLE as well.^10^

Childhood inflammatory myositis, scleroderma and Sjögren disease may be regarded within the spectrum of adult disease because of similarities in pathogenesis between children and adults. The new harmonized classification criteria for inflammatory myopathies proposed by EULAR and ACR were derived and validated in both adult and juvenile cohorts.^11^ However, these criteria have limitations, particularly related to the exclusion of features like calcinosis and certain myositis‐specific autoantibodies, which are more prevalent in children.

The provisional classification criteria for juvenile systemic sclerosis, proposed by PReS, ACR, and EULAR, overlap with the revised adult criteria, but also include additional features relevant to children.^12^ The proposal to develop distinct criteria for juvenile systemic sclerosis that incorporate specific features such as digital ulcers and periungual capillary abnormalities is driven by the aim to improve their relevance for research, diagnostic, and management of systemic sclerosis in children.^12^

Childhood‐onset Sjögren disease is particularly challenging because it is rare and lacks validated classification criteria. Although diagnostic algorithms and scoring systems have been proposed to support diagnosis and classification, these are not yet widely adopted.^13^ The 2016 EULAR/ACR criteria for adult Sjögren disease^14^ do not classify many pediatric cases, often because children exhibit less dryness, the cardinal clinical feature in adults.^15^

Vasculitides present additional classification challenges because of the restriction of certain types of vasculitides to certain age groups. These diseases vary significantly in prevalence and presentation between children and adults and need to be approached in a distinct manner. For instance, giant cell arteritis does not occur in children, whereas Kawasaki disease is virtually exclusively encountered in pediatric populations; therefore, exploring classification criteria across the life course is less relevant for these two conditions. For other types of vasculitides affecting individuals of all ages, the main differences are in relation to the prevalence of various manifestations rather than disease pathogenesis. The EULAR/PRINTO/PReS‐endorsed Ankara 2008 criteria for childhood vasculitides, validated and widely used, provide a basis for accurate classification.^16^ Distinct pediatric classification criteria were proposed for IgA vasculitis (Henoch–Schoenlein purpura), granulomatosis with polyangiitis (GPA), Takayasu Arteritis, and polyarteritis nodosa. However, recent ACR/EULAR adult classification criteria developed from the Diagnosis and Classification of Vasculitis Study (DCVAS) registry highlight the ongoing evolution of classification systems for adult vasculitides.^17^ Interestingly, both the Ankara 2008 and the DCVAS‐derived ACR/EULAR adult criteria had a similar performance in children with GPA,^18^ suggesting potential for harmonization across the lifespan.

Autoinflammatory diseases primarily manifest in childhood and are characterized by recurrent systemic inflammation due to innate immune system dysfunction. The Eurofever initiative has been instrumental in gathering data on autoinflammatory diseases in children, leading to the proposal of shared classification criteria endorsed by both pediatric and adult experts. This collaborative approach underscores the potential for unified criteria that can be applied across age groups, as well as the expansion of large‐scale research initiatives, including clinical trials, facilitating improved disease recognition across the life span, high‐quality research, and progress toward tailored therapies.^19^

## One size does not fit all: the value of expertise sharing and future research needs

Classification criteria should be derived through methodologically sound studies applied to age, sex and gender, and ethnically diverse cohorts with certain RMDs as well as corresponding disease mimickers. Although shared classification criteria across children and adults with similar RMDs would be advantageous in terms of harmonizing research across the lifespan and would support transition of care and access to treatments, the impact of age at onset on RMD manifestations, reflecting both the genetic burden and exposome influences on disease pathogenesis, as well as differences in clinical presentation early compared to later in life, likely determine their performance and clinical usage.

In addition to harmonizing research in RMDs, shared classification criteria could support innovative management strategies, if the disease is similar in children when compared with both younger and older adults. The British Society of Rheumatology endorses evidence and consensus‐based recommendations for management of various RMDs, coproduced by pediatric and adult rheumatologists allowing an across the life course perspective. For conditions with similar pathogenesis, proposing new childhood‐specific classification criteria may not be entirely appropriate, but this has to be balanced against differences in clinical presentation between children and adults with impact on identifying homogeneous disease groups.

Even if a need for better classification criteria is identified, these criteria can only be derived from optimally designed prospective longitudinal cohorts with control groups, including key disease mimickers, robust methodologic approaches, and adequate RMD population representation. This will require collaborative work across pediatric, transition, and adult care specialists, and critical input from patient‐experts of all ages. We identified several challenges for optimizing classification criteria for RMDs across the lifespan and proposed future research strategies, which we included in Table 1.

**Table 1:** Challenges in optimizing the classification of RMDs across the lifespan and future research needs*

Challenges for optimizing classification criteria across the life course	Examples of RMDs for which these challenges are relevant	Future research needs
Lack of correspondence between the diagnostic labels used in children and adults with inflammatory arthritis with similar pathogenesis and clinical presentation	JIA vs adult inflammatory arthritides	Expert consensus studies to review the available literature data to propose and refine the nomenclature used for distinct subcategories of inflammatory arthritis to reflect similarities across pediatric and adult phenotypes
Scarcity of high‐quality studies with geographic representation across the lifespan to define homogeneous RMD clinical phenotypes in children and adults	Lack of studies in JIA in adulthood to enable phenotype correlations with adult inflammatory arthritides; lack of initiatives or studies in SLE, inflammatory myositis, scleroderma, antiphospholipid syndrome, and rare types of vasculitides across the lifespan; recent progress has been achieved in harmonizing the longitudinal data collection across geographically diverse cohorts in some conditions (SLE, myositis, autoinflammatory diseases) but not across the life course	More collaborative, high‐quality, prospective clinical studies with long‐term follow‐up, involving pediatric and adult rheumatologists, and patient populations across the lifespan to define shared clinical phenotypes as well as epidemiologic differences at disease presentation to support future development or refinement of classification criteria; initiation of harmonized national registries linked together across various geographic areas, capturing data and samples from individuals with RMDs across the lifespan
Scarcity of high‐quality studies focused on defining the pathogenesis of RMDs affecting both children and adults	There are almost no studies exploring in parallel the pathogenesis of similar RMDs in children vs adults	High‐quality studies across the lifespan including genetic, molecular, or tissue diagnosis to define commonalities and differences in the disease pathogenesis in children vs adults, and their clinical correlates; linked‐in pediatric and adult RMD‐specific registries
Use of existent classification criteria across the lifespan without being validated in both children and adults	Particularly relevant for rare RMDs, such as antiphospholipid syndrome, some vasculitides, IgG4‐RD, etc, but also for inflammatory arthritides, which are classified completely differently in children vs adults	High‐quality studies to cross‐validate the existent classification criteria in both children and adult cohorts, to evaluate whether there is any need to revise the available criteria
Timely incorporation of advances in medical technologies and discoveries in classification criteria for RMDs across the lifespan	Inclusion of imaging as classification item for Sjögren disease (salivary gland ultrasound) or myositis (magnetic resonance imaging for assessment of muscle inflammation)	Periodic refinement and testing of existent classification criteria to reflect advances in diagnostic tests or incorporate new emerging phenotypes

* IgG4‐RD, IG4‐related disease; JIA, juvenile idiopathic arthritis; SLE, systemic lupus erythematosus; RMDs, rheumatic diseases.

## Conclusions

Classification criteria will require periodic review to incorporate advances in medical technologies and discoveries, as well as changes in research priorities to facilitate early disease classification and timely management interventions, for the overall aim to improve disease outcome and preserve quality of life at any age. We advocate for good performance classification criteria, reflecting shared pathogenic mechanism across age‐distinct RMD phenotypes, which should be feasible and easy to implement. This will facilitate adequate dissemination of knowledge related to various disease processes, as well as innovation in RMD management, ultimately aiming to support wider and fairer access to research for diverse populations of all ages.

## AUTHOR CONTRIBUTIONS

All authors were involved in drafting the article or revising it critically for important intellectual content, and all authors approved the final version to be published.

## Supporting information


Disclosure form
